# Total Hip Arthroplasty in Systemic Lupus Erythematosus: A Systematic Review

**DOI:** 10.1155/2015/475489

**Published:** 2015-07-08

**Authors:** Ian W. Kennedy, Wasim Khan

**Affiliations:** ^1^Department of Trauma and Orthopaedics, Southern General Hospital, 1345 Govan Road, Glasgow G51 4TF, UK; ^2^University of Glasgow, Glasgow G12 8QQ, UK; ^3^University College London Institute of Orthopaedics and Musculoskeletal Sciences, Royal National Orthpaedic Hospital, Stanmore HA7 4LP, UK

## Abstract

The prognosis of systemic lupus erythematosus (SLE) has greatly improved in recent years, resulting in an increased number of patients reporting musculoskeletal complications such as osteonecrosis of the femoral head. Total hip arthroplasty (THA) can be utilised to alleviate the pain associated with this; however postoperative outcomes in patients with SLE are uncertain. A systematic review of the literature was conducted to identify articles presenting results of THA in SLE, and nine suitable papers were found. All papers were level IV evidence. Pooling the results, a total of 162 patients underwent 214 total hip arthroplasties. Mean follow-up was 72.5 months. The mean Harris Hip Score improved from 45.5 preoperatively to 88.6 and last follow-up. Seventeen percent of patients experienced at least one complication. Superficial wound infection occurred in 3.3%. Revision was required in 2.8% of cases. The mortality rate was 18.5% however no deaths were attributable to undergoing THA. Given the paucity of data present in the literature, more studies are required to adequately assess the postoperative outcomes of THA in patients with SLE, particularly complication rates.

## 1. Introduction

Systemic lupus erythematosus (SLE) is a chronic autoimmune systemic disease with a range of clinical manifestations. It is characterised by the production of antinuclear autoantibodies and occurs most commonly in young women [1]. Survival in new onset SLE has improved greatly with more effective management options. Prior to 1955, less than 50% of patients survived five years after diagnosis; now, 10 year survival exceeds 90% [2]. This has resulted in an increased rate of reported musculoskeletal conditions, including osteonecrosis and inflammatory arthritis. Although the arthritic changes seen in SLE are not typically erosive and destructive as can be seen in rheumatoid arthritis (RA), features of both SLE and RA can coexist in some patients [3]. Osteonecrosis of the femoral head occurs in around 10% of SLE patients [4] and can be secondary to vasculitis or long-term corticosteroid use [5]. Some patients may report that osteonecrosis causes a greater reduction in their quality of life than the underlying systemic disease [3]. Following collapse of the femoral head, pain and reduced function can be severe, warranting surgical intervention. Initial options include femoral osteotomy [6], core decompression [7], and bone grafting [8]. Such procedures are not always successful, and so total hip arthroplasty (THA) is often required [9]. Although THA has been shown to provide reliable symptomatic relief and functional improvement in osteoarthritis [10], the postoperative outcomes and complication rates in SLE patients are less well understood. This is mainly due to the limited number of small reports within the literature. There are a number of fundamental differences between SLE patients and the typical THA case that make it difficult to predict postoperative outcomes in the former group based on evidence for the latter. Firstly, many SLE patients will be less than 40 years of age at the time of surgery and so THA needs to be undertaken with the aim of providing long term positive clinical outcomes to avoid repeated revision surgeries. Younger patients also have higher activity levels, leading to greater revision rates [11]. Furthermore, given the frequent use of immunosuppressives and corticosteroids preoperatively, it has been postulated that SLE patients will have a higher rate of prosthetic joint infection [3] which carries significant morbidity. High dose steroids may also lead to large areas of osteonecrosis extending into the calcar, leading to inadequate support for the femoral component [12]. This systematic review therefore sets out to examine the current literature regarding THA in SLE patients with the aim of determining clinical outcomes and complication rates.

## 2. Methods

Four databases were searched (Medline, Embase, PubMed, and The Cochrane Library) on 19th June 2014. The following search algorithm was used: ((“systemic lupus erythematosus” OR “SLE” OR “lupus”) AND (“total hip arthroplast⁣^*∗*^” OR “total hip replacement⁣^*∗*^” OR “hip replacement⁣^*∗*^” OR “hip arthroplast⁣^*∗*^” OR “THA”)). No limit was set for date of publication and only English-language articles were included. Duplicates were removed before conducting a hand search of relevant article bibliographies to identify any additional studies. The abstracts of all articles were reviewed to identify potentially relevant studies. Review articles, conference papers, and those which did not involve THA in SLE patients' were excluded. The full-texts of all remaining studies were then examined to determine eligibility. Articles were deemed not eligible if (1) results of other procedures, such as hemiarthroplasty, were combined with THA and not reported separately; (2) SLE patients' were combined with other non-SLE conditions, such as rheumatoid arthritis, and not reported separately; and (3) insufficient clinical data was presented (i.e., no hip scores, complications, revision, or mortality rates). Inclusion and exclusion criteria are presented in Table 1. The following data was obtained from the final studies, where possible: patient characteristics, sample size, length of follow-up, implant type (i.e., cemented or uncemented), hip scores, complications, mortality, and revision rates. Extracted data was collated on a specifically designed spreadsheet. Continuous variables were reported as means +/− standard deviations from the mean. Categorical variable data was reported as a frequency with percentages.

## 3. Results

**Figure 1:**
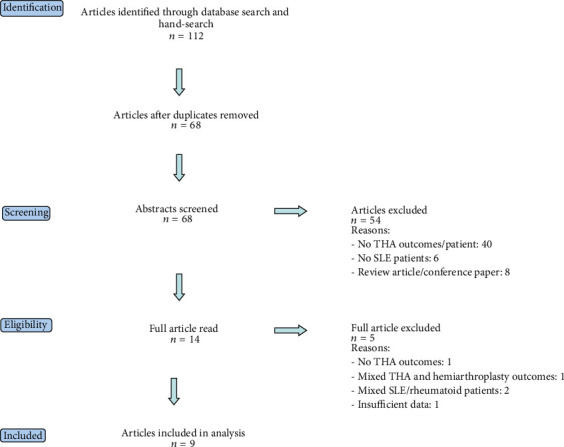
Flow diagram of included studies.

Our literature search identified 68 articles after duplicates were removed. Following review of all abstracts, 54 articles were excluded for not meeting the eligibility criteria. The full-texts of the remaining 14 articles were examined, and nine were deemed suitable for inclusion in this systematic review.  Figure 1 outlines our literature search process.

All articles were Level IV evidence, published from 1987 to 2013. There were a total of 162 patients who underwent 214 total hip arthroplasties. Gender was documented in all but one study, with 85% of participants being female, 15% male. Mean age at surgery was 36.6 years (standard deviation, SD, 5.2). Mean follow-up was 72.5 months (SD 35.4). All articles except one specified whether implants were cemented or uncemented, with 88.1% being uncemented, 4.3% being cemented, and 7.6% being hybrids (e.g., uncemented acetabular component, cemented femoral stem).

Seven articles assessed functional outcomes with the Harris Hip Score (HHS) and two used the Mayo Clinic Hip Score (MCHS). Higher scores indicate better outcomes in both methods, with a maximum score of 100 for the HHS and 80 for the clinical component of the MCHS. For the HSS, there was a mean increase from 45.5 (SD 8.9) preoperatively to 88.6 (SD 5.1) postoperatively. A score of 80–90 correlates to a “good” outcome and 90–100 to “excellent.” The preoperative MCHS was only documented in one paper (10.4). Postoperatively, the mean score improved to 75.8 (SD 0.4).

Complication rates were documented in five papers. A mean rate of 17.3% was found in these studies. For the remaining four articles, two did not record complication rates and two did not provide absolute values; one stated that the rate was “not low.” Revision rates were documented in eight papers, producing a rate of 2.8%. Mortality rates were documented by four papers. In this group, 18.5% of patients had died by last follow-up. There were no deaths attributable to surgery. Individual article result summaries are displayed in Table 2.

## 4. Discussion 

The prognosis of SLE patients has improved greatly in recent times; 5 year survival in 1955 was approximately 50% in 1955 compared to 10 year survival of >90% at present [2]. Improving quality of life by managing musculoskeletal complications is therefore of great value to these patients. Total hip arthroplasty has been shown to reliably manage pain and disability secondary to osteoarthritis [10]. However postoperative outcomes such as hip scores and complication rates are less well understood in SLE patients. This can be attributed to a limited number of research articles within the literature, small sample sizes, and the absence of a comparator group. This systematic review, therefore, aims to examine the current literature pertaining to total hip arthroplasty in patients suffering from systemic lupus erythematosus.

There are limitations to this study. Firstly, all studies were level IV evidence [21], thus limiting the reliability of the results. There were also a variety of variables within the studies. For example, a number of different hip implants were used, both cemented and uncemented, which could make the results more generalizable, but would introduce discrepancies between studies. Two different hip scoring methods were also used (Harris Hip Score and Mayo Clinic Hip Score) which reduced the sample sizes available for each respective group. Additionally, the collection and reporting of complication rates were not explained by the authors' and so it is difficult to determine whether only surgical complications or a complete list were documented, which may explain the range of 1.7% [9] to 48.8% [19]. Finally, there was no complete set of outcomes for all articles; for example, mortality rates were only documented by four authors [13, 17, 19, 20] and complication rates by five [9, 13, 16, 18, 19]. Strengths of this study are the sample size of 214 total hip arthroplasties and length of follow-up (mean 72.5 months).

The Harris Hip Score (HHS) was the most commonly utilised scoring method and there was a significant postoperative improvement, with a mean of 88.6 (80–90 constitutes a “good outcome”). As the HHS assesses pain and function, this suggests that SLE patients will benefit considerably from THA. This is particularly important given that pain is the commonest reason for undergoing THA [22]. The Mayo Clinic Hip Score (MCHS) was utilised by two authors. This scoring method has two components: firstly a clinical score (best score = 80) which tests similar areas to HHS, such as walking distance and use of walking aids, and secondly a radiographic score (best score = 20) which assesses for issues including component migration [23]. However neither paper provided a mean radiographic score, and so only the clinical score was available. The mean clinical score of 75.8 is nonetheless indicative of an excellent functional outcome. It would, however, have been beneficial for radiographic scores to be presented alongside this as higher rates of component loosening in osteonecrosis compared to osteoarthritis have been reported elsewhere [24]. It is also important to note that limited long term data exists regarding hip scores post-THA in SLE patients at present and so further studies are required to assess this [9].

Eighty eight percent of hip replacements in these studies were uncemented. Orban et al. [25] hypothesised that cemented prosthetics would produce more favourable results due to corticosteroids diminishing bone quality and therefore reducing the quality of fixation achievable with uncemented implant. This does not seem to be borne out in the above studies, although there was no comparison between cemented and uncemented THA to adequately assess this. Additionally, Chen and colleagues [20] presented several advantages to using uncemented THA in this cohort, including avoiding cementing complications. They also recommend utilizing autologous bone graft from the femoral head to augment bone stock.

A revision rate of 2.8% was found for the pooled results of eight papers. In their review of THA for femoral head osteonecrosis, Johannson et al. [26] found an overall revision rate of 13%. Although this represents a substantially greater frequency than was found in this study, the mean length of follow-up was longer in those papers reviewed by Johannson, and so it is likely that a higher revision rate would also be present with long-term follow-up of SLE patients. Given the young age at which they may undergo hip replacement, greater demands are often placed on the joint replacement as patients return to athletic activities, with resulting higher revision rates [11]. An accurate analysis of whether SLE patients are at greater risk of requiring repeated revision surgery through long-term follow-up is therefore necessary. Johannson's review also demonstrated a reduction in revision rates for patients operated on after 1990, which correlated with the use of newer hip replacement designs and more frequent use of uncemented implants. There was no link between revision rates and time in the nine papers presented in this systematic review; however the preference for using uncemented implants without any obvious negative effect on revision rates is consistent with the aforementioned evidence. As already noted, further analysis is warranted to truly assess this.

Complication rates were highly variable between studies. On average, 17.3% of patients experience at least one complication. The figures presented in each paper are difficult to interpret, however, due to the absence of a comparator group in these studies. This prevents a useful comparison between the complication rates of THA in SLE patients and those with other pathologies, such as osteoarthritis. Kang et al. [13] did note that their rate of 11.1% was higher than the department average of 3.1%, but this did not represent a matched cohort. There were several dislocations reported; Shigemura et al. [14] documented an incidence of 14.3%. A prior study [27] demonstrated a higher dislocation rate in femoral head osteonecrosis when compared to osteoarthritis. It has been suggested that this may be due to greater soft tissue laxity in these patients, resulting in a greater range of motion and less femoral head constraint which ultimately predisposes to dislocation. Specific surgical alterations, such as utilizing a larger femoral head and capsular repair, may reduce the likelihood of dislocation in these patients [14].

Due to the frequent use of corticosteroids and immunosuppressant's in SLE, it has been suggested that there may be a higher prosthetic joint infection rate compared to patients not receiving such therapies [3]. In the studies which discussed complications, there were no documented cases of deep infection. However there were six patients (3.3%) with superficial wound infections. Peel and colleagues [28] found in their analysis of prosthetic joint infection that 5.6% of patients will develop a superficial wound infection without progression to deep infection, and so the studies within this systematic review actually had a lower infection rate. Low [18] suggested that their series had low infection rates due to all patients being in remission at the time of operation, and so they did not receive any steroids perioperatively. They felt that higher complication rates would be present if this was not the case. Kang also stated that in addition to low disease activity, SLE patients were given more antibiotics than their general cases [13]. Conversely, Hanssen did not find any correlation between complications and administration of corticosteroids at the time of operation [19]. It has been postulated that the link between systemic steroids and prosthetic joint infection is in part due to impaired wound healing, leading to the introduction of infective organisms [29]. Equally, it is difficult to differentiate the effect of the underlying condition from the immunosuppressive therapy when considering infection rates [28]. The disparity between infection rates found in the studies of this systemic review and results from other papers further stresses the need for more robust evidence on joint replacement in SLE.

Similar to complications, there was a wide range of mortality rates, ranging from 12.5% [13] to 30.4% [19]. This seems high when the mean age at surgery was 36.6 years; however all authors stated that there were no deaths secondary to undergoing THA and these deaths occurred >12 months postoperatively. The cause of death was highly variable, including intracranial bleed, sepsis, and heart failure. Equally, many of the papers in this study were published in the 1990's, at which point there were significantly higher mortality rates in SLE patients due to less effective treatment options [2]. This is consistent with Kang and colleagues [13] 2013 article having a mortality rate of 12.5% compared to 30.4% in Hanssen's [19] 1987 study.

## 5. Conclusion 

This systematic review demonstrated that SLE patients who undergo THA can expect high hip scores postoperatively, indicating good function and pain relief. There were also no deaths attributable to THA and revision rates were relatively low. Complication rates are highly variable, however, and so the risks of this procedure are difficult to establish at present. In particular, although infection rates were not high in these studies, the use of systemic steroids in patients requiring joint replacement is a cause for concern due to their effect on wound healing and infection risk. More studies are therefore required to adequately assess the safety of hip replacement in this patient group and also to quantify long term outcomes, ideally through prospective studies with a comparator group. In view of the small number of cases and publications, the national registry needs to better collate these patients so more meaningful data about implants and outcome can be achieved that would guide future management and implant choice [30, 31].

## Figures and Tables

**Table 1:** Inclusion and exclusion criteria for articles.

Inclusion criteria	Exclusion criteria
English-language articles	Review articles
Any date of publication	Conference abstracts
Presented outcomes of THA in SLE patients	Not involving THA in SLE patients
	Other procedures, such as hemiarthroplasty, combined with THA results
	SLE patients combined with other non-SLE conditions (e.g., rheumatoid arthritis)
	Insufficient clinical data (i.e., no hip scores or complication, revision or mortality rates)

**Table 2:** Total hip arthroplasty outcomes from nine studies. No recorded value is indicated by “NR.”

Paper	*N* patients (THA)	Gender (F = Female, M = Male)	Mean age (years)	HHS pre-/postoperative	MCHS pre-/postoperative	Complications (%)	Revision (%)	Mortality (%)	Notes
Kang et al. 2013 [13]	24 (28)	19 F, 5 M	38.8	33.0/84.3	NR	11.1 (i) 1 culture negative prolonged wound drainage, 1 culture positive prolonged wound drainage managed with antibiotics, 1 urinary tract infection	0	12.5 (i) 1 intracranial bleed at 18 months, 1 pneumonitis and heart failure at 24 months, 1 myocardial infarction at 60 months.	Complication rate higher than unit average of 3.1%. Rate of 11.1% is for 27 THA; authors do not supply reason for omitting 1 THA.

Issa et al. 2013 [9]	44 (60)	37 F, 7 M	42	44/87	NR	1.7 (i) 1 lupus pleuritis	1.7 (i) 1 aseptic loosening	NR	

Shigemura et al. 2013 [14]	11 (14)	9 F, 2 M	35.2	37.4/94.5	NR	NR (i) include 2 dislocations and 1 peroneal nerve palsy	14.3 (i) 1 polyethylene insert dislodged, 1 osteolysis and polyethylene insert dislodged	NR	Complication rates “not low,” however no absolute value provided.

Ito et al. 2007 [15]	20 (25)	NR	NR	NR/84.3	NR	NR	4.0 (i) 1 aseptic loosening	NR	Majority of data in study was a combination of THA and bipolar hemiarthroplasty, limiting results.

Chen et al. 1999 [16]	14 (17)	13 F, 1 M	29.8	50.7/95.5	NR	NR (i) included 1 superficial infection, 1 dislocation, 1 groin pain and 3 polyethylene wear	0	NR	No absolute complication rates documented.

Brinker et al. 1994 [17]	6 (8)	5 F, 1 M	38.9	53.1/83.5	NR	NR	0	16.7 (i) cause not stated	Subset of patients from study examining THA in osteonecrosis of multiple aetiologies.

Low et al. 1991 [18]	6 (10)	5 F, 1 M	31.2	NR	10.4/75.5	20 (i) 1 transient sciatic nerve palsy, 1 aseptic loosening	NR	NR	

Hanssen et al. 1987 [19]	23 (29)	20 F, 3 M	44	NR	NR/76.0	44.8 (i) 5 delayed wound healing, 4 superficial wound infections, 2 SLE exacerbations, 2 wound haematomas, 2 transfusion reactions, 1 thrombocytopenia, 1 psychosis, 1 dislocation, 1 urinary tract infection	0	30.4 (i) 1 sepsis, 1 chronic renal failure, 1 stroke, 1 heart failure, 1 intrapulmonary haemorrhage, 1 acute SLE flare, 1 cause unknown.	No pre-operative MCHS score. 22 complications occurred in 13 THA.

Chen and Lin 1987 [20]	14 (23)	13 F, 1 M	32.8	54.9/91.3	NR	(i) 8.7 (ii) 1 dislocation, 1 acetabular component loosening	8.7 (i) 1 dislocation, 1 acetabular component loosening	14.3 (i) 2 SLE and infection	

## Abbreviations

THA: Total hip arthroplasty
SLE: Systemic lupus erythematosus
HHS: Harris Hip Score
MCHS: Mayo Clinic Hip Score
SD: Standard deviation.
